# *IDH* Mutations Are Potentially the Intrinsic Genetic Link among the Multiple Neoplastic Lesions in Ollier Disease and Maffucci Syndrome: A Clinicopathologic Analysis from a Single Institute in Shanghai, China

**DOI:** 10.3390/diagnostics12112764

**Published:** 2022-11-11

**Authors:** Chunyan Chen, Jian Li, Ting Jiang, Juan Tang, Zhichang Zhang, Yanli Luo, Xinpei Wang, Keyang Sun, Zhiming Jiang, Juan Zhou, Zhiyan Liu

**Affiliations:** 1 Department of Pathology, Shanghai Sixth People’s Hospital Affiliated to Shanghai Jiao Tong University School of Medicine, Shanghai 200233, China; 2 Department of Pathology, Peking University Shenzhen Hospital, Shenzhen 518036, China; 3 State Key Laboratory of Chemical Oncogenomics, Peking University Shenzhen Graduate School, Shenzhen 518055, China; 4 Department of Orthopaedics, Shanghai Sixth People’s Hospital Affiliated to Shanghai Jiao Tong University School of Medicine, Shanghai 200233, China; 5 Department of Pathology, School of Basic Medical Sciences, Cheeloo College of Medicine, Shandong University, Jinan 250012, China

**Keywords:** isocitrate dehydrogenase, Ollier disease, Maffucci syndrome, cartilage tumour, extraskeletal neoplasms, haemangioma, paediatric ovarian tumour

## Abstract

Background: This study aims to investigate isocitrate dehydrogenase gene mutations in patients with the non-hereditary skeletal disorders of Ollier disease and Maffucci syndrome, particularly in the extraosseous tumours. Methods: A total of 16 tumours from three patients with Ollier disease and three patients with Maffucci syndrome were collected. Sanger sequencing was applied to determine the hotspot mutations of *IDH1* and *IDH2* genes in multiple neoplastic tissues. Results: A majority of the tumours displayed an *IDH1* mutation (p.R132C in 11 tumours including the paediatric ovarian tumour from one patient with Ollier disease, 4 cutaneous haemangiomas from three patients with Maffucci syndrome, 5 enchondromas and 1 chondrosarcoma; p.R132H in 2 cartilaginous tumours from one patient). Conclusions: *IDH1* mutations were demonstrated in multiple cartilaginous tumours and extraskeletal neoplasms in this case series. Specifically, identical *IDH1* mutations were confirmed in the separate lesions of each patient. These results are in concordance with findings that have been reported. However, here, we additionally reported the first case of Ollier disease with an ovarian tumour, which harboured the identical *IDH1* mutation with the corresponding cartilaginous tumour. We further provided evidence that *IDH* mutations are the potential genetic links among the multiple neoplastic lesions of Ollier disease and Maffucci syndrome.

## 1. Introduction

Enchondromatosis is a syndrome hallmarked by a benign cartilage-forming tumour within the bones. The most common subtypes are non-hereditary Ollier disease and Maffucci syndrome [1,2], mainly involving the short tubular bone and long bone of the limbs asymmetrically, and the latter is accompanied by simultaneous haemangioma of the dermis or subcutis or internal organs. Patients with Ollier disease and Maffucci syndrome often manifest in early childhood, and surgical intervention is usually needed to correct deformities in the process of growth and development. However, the incidence of malignant transformation of enchondromas to chondrosarcoma is high; it ranges from 5% to 50% in Ollier disease and could be as high as 53% in Maffucci syndrome [1,3,4]. In addition, both disorders have an increased risk of extraosseous malignant tumours, and particularly of ovarian juvenile granulosa cell tumours and central nervous system gliomas [2]. 

As is known, the hallmark genetic abnormality of enchondroma associated with Ollier disease and Maffucci syndrome is the somatic point mutations on isocitrate dehydrogenase (*IDH*) genes, *IDH1* and *IDH2* [5,6,7,8]. Tumours in separate sites of one individual display identical mutations. However, the genetic correlation among multiple tumours especially in extraosseous malignancies associated with the two disorders remains uncertain.

Herein, we retrospectively reviewed three cases of Ollier disease and three cases of Maffucci syndrome. *IDH1* and *IDH2* gene mutations were performed in a series of tumours from the 6 patients, including 1 ovarian tumour, 4 vascular tumours and 11 cartilage tumours. The specific *IDH* mutations within cartilage tumours and non-cartilage tumours of Ollier disease and Maffucci syndrome were compared.

## 2. Case Report

### 2.1. Case Selection

Three patients with Ollier disease and three patients with Maffucci syndrome were selected from Shanghai Sixth People’s Hospital Affiliated to Shanghai Jiao Tong University School of Medicine during the period from 2016 to 2020. Ethical approval was given by the medical ethics committee of this hospital. The information of the patients was collected, including clinical symptoms, imaging reports (X-ray, CT or MRI) and follow-up data. All haematoxylin and eosin stain (H&E) sections were confirmed by C.Z. and J.Z. A total of 16 tumours and paired normal tissues were sampled from the six patients for the detection of *IDH1* and *IDH2* mutations by Sanger sequencing. Five enchondromas, one chondrosarcoma and one ovarian tumour were from the three patients with Ollier disease. Four enchondromas, one chondrosarcoma and four vascular tumours were from the three patients with Maffucci syndrome.

### 2.2. Clinical Data

**Figure 1:**
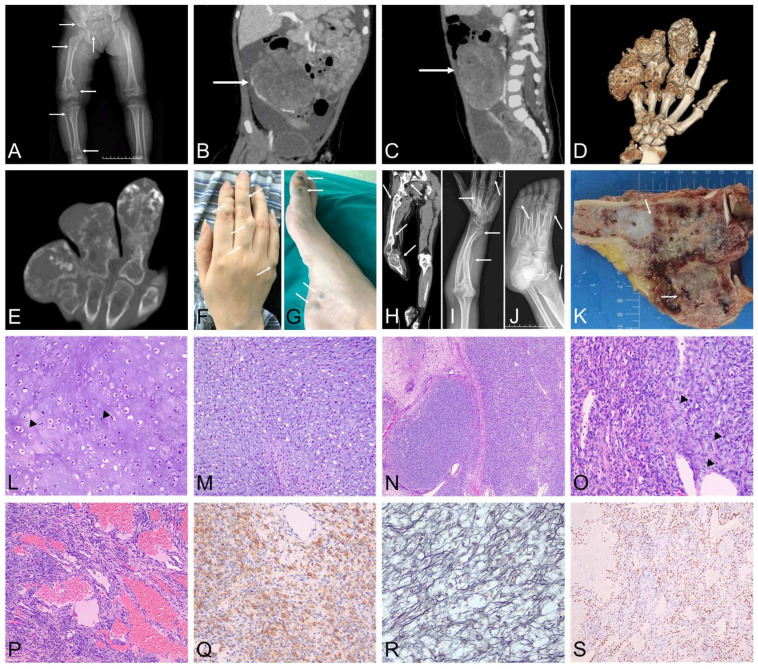
(**A**) Spotted bone destruction of the right upper femur, tibial metaphysis, iliac and pubis was shown by X-ray (arrow). (**B**,**C**) A huge soft tissue mass in pelvis could be observed from the MRI (arrow). (**D**,**E**) Multiple expansive bone destruction was shown in the right little finger; ring finger; middle finger; the third, fourth and fifth metacarpal bones; and the distal ulna by three-dimensional reconstruction and CT. (**F**,**G**) Multiple purplish blue subcutaneous nodules were found in the hands and feet, protruding on the surface of the skin (arrow). (**H**) Multiple bone destruction was seen in bilateral ilium, right acetabulum, left ischium, bilateral femur, right tibia, fibula and calcaneus by PET-CT (arrow, still image). (**I**,**J**) A curved deformity of the left radius and ulna, and eccentric low-density shadow of multiple short tubular bones of left hand and foot and the left distal tibia were presented by plain X-ray (arrow). (**K**) Well-differentiated hyaline cartilage (arrow, up) and dark red sticky area involving the surrounding soft tissue were seen in the cut section of the right proximal tibial tumour (arrow, down). (**L**) Active chondrocytes were distributed in clusters, and atypical binucleate cells (arrow) could occasionally be seen (Hematoxylin-eosin staining, ×200). (**M**) Chondrocytes with severe atypia could be demonstrated in the sarcomatous area, no more cartilage lacunae could be found (Hematoxylin-eosin staining, ×100). (**N**) Lobular-patterned ovarian sex cord-stromal tumour with mucinous degeneration was seen at low power (Hematoxylin-eosin staining, ×40). (**O**) The tumour cells had obvious nucleoli and light eosinophilic cytoplasm, and mitotic figures (arrow) were easy to find at high power (Hematoxylin-eosin staining, ×200). (**P**) Solid spindle cell and cavernous haemangioma-like areas of Maffucci syndrome (Hematoxylin-eosin staining, ×100). (**Q**) Positive immunoreaction with α-inhibin was seen in the cytoplasm in ovarian tumour (EnVision, ×200). (**R**) Reticular fibres were confirmed in the ovarian sex cord-stromal tumour (Reticular fibre staining, ×200). (**S**) Positive immunoreaction with ERG was observed in the nucleus of the haemangioma (EnVision, ×100).

All patients had no corresponding family history. The sites of the tumours ranged from one or several to dozens of lesions at most (Table 1). The cases of Ollier disease included two females and one male, and the age at diagnosis was mainly at young age (Table 1, case 1–3). In case one, the CT revealed lytic bone destructions in the right tibial metaphyseal, superior femur, ilium and pubis (Figure 1A), and the MRI showed a huge mass in the pelvis (Figure 1B,C). In case two, the patient presented with a nodular lesion of the right middle finger at 3-years-old. Eccentric low-density shadows were shown gradually in the proximal segment of the right middle finger and the right third metacarpal bone during the 13 years follow-up by X-rays. In case three, a swelling was found in the right finger at 7-years-old. The finger gradually enlarged in the following 48 years. Surgery was rendered because the nodule grew fast at the age of 55. Multiple nodular lesions with eccentric expansive growth and irregular calcification were demonstrated by imaging (Figure 1D,E), which locally penetrated the bone cortex and extended into the surrounding soft tissue.

Maffucci syndrome (cases 4–6) included two females and one male (Table 1). In case four, the patient presented with a swelling of the right finger and a left ulnar lesion at the age of three. The left ulnar nodule was proven to be enchondroma histologically by excision at the age of six. In the following 14 years, multiple intraosseous eccentric masses were presented gradually on his right fingers, accompanied by multiple subcutaneous soft tissue masses. Case five began to have nodules in her hands and feet when she was only two–three-years-old. In the following 33 years, extensive purple-blue skin masses appeared subsequently in the hands, feet, limbs, shoulders and back (Figure 1F,G). The patient showed rapid progression of swelling and pain in her right knee for about half a year before her consultation. The PET-CT scan showed multiple expansive bone destructions with irregular calcification and an accompanying soft tissue mass surrounding the right proximal tibia lesion (Figure 1H). In case six, a deformity of the left radius and ulna was identified at the age of six. An X-ray analysis showed a curved deformity of the left radius and ulna, and eccentric low-density shadow of multiple short tubular bones of the left hand and foot and the left distal tibia (Figure 1I,J). MRI showed multiple high-density nodules in the left plantar and medial subcutaneous.

Benign cartilaginous lesions from six patients underwent curettage selectively. Resection was performed in 2 cartilaginous tumours which manifested local malignant transformation to chondrosarcoma from 2 patients, respectively. The ovarian tumour in case one was resected. Four subcutaneous nodules were resected from three cases of Maffucci syndrome.

### 2.3. Pathological Examination

The gross appearance of the chondrogenic tumour tissue was grey, translucent and cartilage-like. In case three: obvious deformity and swelling of the phalanx was observed in the half palmar resection specimen; multiple nodular cartilaginous lesions destroyed local bone cortex and invaded into the surrounding soft tissue. In case five: a translucent cartilaginous mass in the medullary cavity extending from the tibial bone end towards the metaphysis was demonstrated in the resected specimen of the right proximal tibial; a dark red jelly-like tumour with an irregular margin penetrated into the bone cortex and the surrounding soft tissue (Figure 1K).

All enchondromas showed a similar lobulated structure at low power, high cellularity at high power, accompanied by mild atypia and binuclear cells occasionally (Figure 1L). In the chondrosarcoma of 2 cases, atypical tumour cells with extensive mucinous degeneration of the cartilage matrix infiltrated into the cortex and soft tissue (1M). The ovarian tumour of case one consisted of primitive and immature tumour cells within the mucinous degeneration of interstitial components distributing in a flaky pattern with fibrous separation at low power, (Figure 1N). The tumour cells were ovate to elongated with light eosinophilic cytoplasm and had obvious nucleoli at high power (Figure 1O). Three cases with Maffucci syndrome presented a mixed spindle cell haemangioma and cavernous haemangioma (Figure 1P).

### 2.4. Immunohistochemical Features and Special Staining

The ovarian tumours in Ollier disease showed an immunoreaction with α-inhibin (Figure 1Q), and reticulin staining displayed tumour cells surrounded by reticular fibres (Figure 1R). The spindle cell haemangioma and cavernous haemangioma in Maffucci syndrome were positive for ERG (Figure 1S).

### 2.5. Molecular Features 

**Figure 2:**
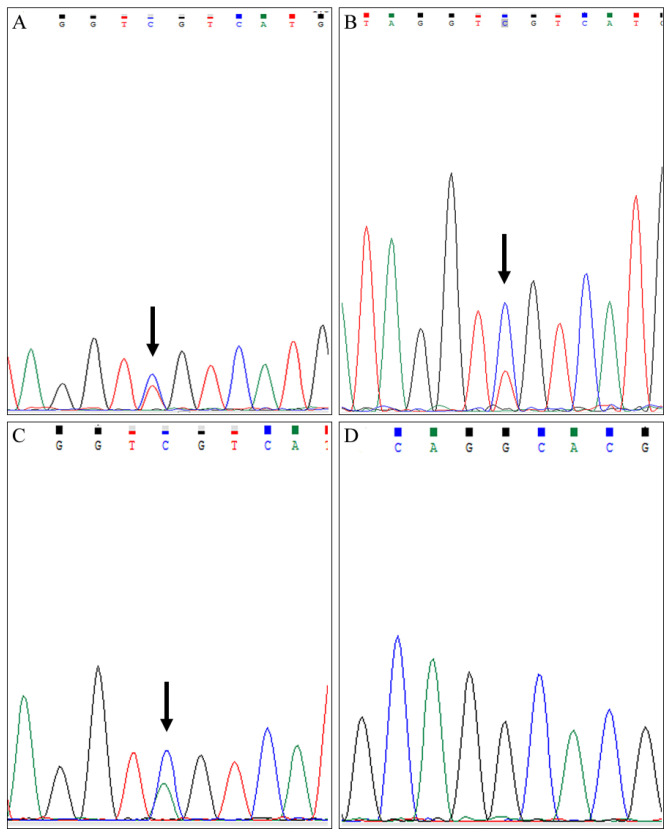
(**A**) *IDH1* mutation in ovarian tumour: p.R132C (c.394C > T) (indicated by arrow). (**B**,**C**) *IDH1* mutations in cartilage tumours: p.R132C (c.394C > T) (indicated by arrow) and p.R132H (c.395G > A) (indicated by arrow). (**D**) *IDH2* mutation: wild type.

*IDH1* and *IDH2* mutations were detected by Sanger sequence. The molecular features were summarised in Table 2. *IDH1* mutations were discovered in most cartilage tumours except for three enchondromas. All 4 haemangiomas detected an *IDH1* mutation. The ovarian tumour from a patient with Ollier disease was shown to carry the R132C *IDH1* mutation. Multiple separate tumours in one individual shared identical *IDH1* mutations. The *IDH1* mutation variant of three patients with Ollier disease were p.R132C, p.R132C and p.R132H, respectively, and that of three patients with Maffucci syndrome was all p.R132C (Figure 2). The detection rate of *IDH1* mutations in cartilage tumours was 73% (8/11). All the non-chondrogenic tumours were detected to have identical hotspot mutation locations of *IDH1* with the corresponding cartilage tumours. There were no *IDH2* mutations detected.

### 2.6. Prognosis

Follow-up information as showed in Table 1 was available for all patients by radiographic examination and telephone interviews. In case two, no relapse or new lesions were demonstrated after surgery up to now. The remaining five patients were alive without recurrence and distant metastasis.

## 3. Discussion

Ollier disease and Maffucci syndrome are characterized by multiple enchondromatosis, and the phenotypic difference between them is mainly the existence of vascular anomalies [1,9]. Maffucci syndrome may show more severe mesodermal dysplasia in that the extraskeletal areas are often involved. The cartilaginous skeletal dysplastic are easy to be identified; however, minimal soft tissue haemangiomas are hard to recognize initially. Therefore, Maffucci syndrome is usually mistaken for Ollier disease in the early stage. About 75% of those patients are diagnosed before the age of 20, and about 45% develop symptoms before the age of six [2,3]. All of our 6 patients presented symptoms before puberty, which were consistent with the previous studies. The incidence of secondary chondrosarcoma associated with Maffucci syndrome and Ollier disease is much higher than that in solitary enchondromas (3). As presented in our group, two cartilaginous tumours developed into chondrosarcomas, which were located in the short and long tubular bone, respectively. However, it is unnecessary to distinguish these two disorders from the clinical perspective; both of them need a lifetime follow-up by imaging after diagnosis. A timely surgical intervention is essential whenever the tumour has a tendency of malignant transformation.

*IDH1* and *IDH2* genes are located on the long arms of chromosome 2 and chromosome 15, respectively [5,6,7,8]. It was reported that *IDH* mutations are identified in 81% of patients with Ollier disease and 77% of patients with Maffucci syndrome. The IDH mutation rate of benign cartilaginous tumours is about 87%, and that of vascular lesions is about 70%. In the current study, Sanger sequencing was used to detect the mutations of *IDH1* and *IDH2* in the 16 tumours of six patients. The mutation rate of *IDH1* in cartilaginous tumours was about 73% (8/11), which is slightly lower than reported. All 4 haemangiomas were confirmed to harbour a *IDH1* mutation. Since the mutant allele could be present as low as 1% (8), which may be below the detection level of the targeted mutation analysis, the true frequency of *IDH1/2* mutation in enchondroma could be underestimated. Saiji et al. reported the mutation rate of *IDH* was 100% in 13 enchondromas from eight patients with Ollier disease using next generation sequencing (NGS) [10]. The difference in methods may explain the lower occurrence (73%) of *IDH* mutations found in our current study, as NGS captures a broader spectrum of mutations than Sanger sequencing.

*IDH* mutations in enchondromatosis associated with Ollier disease most frequently involve Arginine 132 (R132) in exon 4 of *IDH1*, while the *IDH2* gene mutation (mainly p.R172S) appears to be rarely affected. In comparison with Ollier disease, enchondromas and spindle-cell haemangiomas in Maffucci syndrome are characterized by the exclusive predilection of *IDH1*. The mutation variants include mostly R132C, and rarely R132H, R132G. Here, we reported an R132C mutation of *IDH1* in two cases of Ollier disease and three cases of Maffucci syndrome, and R132H in the third case of Ollier disease. All the segregated extraskeletal non-cartilaginous tumours were found to possess the identical *IDH1* mutations with corresponding cartilage tumours. There were no *IDH2* mutations found. 

*IDH1/2* plays important roles in the tricarboxylic acid cycle by converting isocitrate to α-ketoglutarate (α-KG) [11]. The mutations of *IDH1/2* make the mutated enzyme catalyse α-ketoglutarate (α-KG) to (D)-2-hydroxyglutaric acid (D-2-HG) [12,13,14,15], which has already been confirmed that it could induce multiple enchondromas during bone formation through promoting chondrocyte differentiation and inhibiting osteogenic differentiation of mesenchymal stem cells [16,17]. As presented here, *IDH1* mutations were identified both in six enchondromas and two secondary chondrosarcomas. However, these mutations are merely an early driver of tumourigenesis in cartilage tumour formation, and further malignant transformation into chondrosarcoma may require additional genetic events [7,18]. Furthermore, the excessive intracellular accumulation of D-2-HG impairs histone demethylation, and leads to aberrant epigenetics changes of DNA methylation and abnormal cellular differentiation [7,19,20,21,22,23]. Therefore, *IDH1/2* mutations are believed to contribute to the development of a variety of tumours.

Ollier disease and Maffucci syndrome share the same phenotypic characteristics: (1) non-hereditary; (2) related tumours with asymmetrical polyostotic distribution, suggesting the genetic characteristic of somatic mosaic mutations. Multiple tumours in the same individual possess identical *IDH1* mutations, and a very low frequency of *IDH* mutant protein is observed in normal tissue in patients with Ollier disease and Maffucci syndrome [6,7,8]. Those reports further confirmed that *IDH* mutations occur in a somatic mosaic pattern. The identification of the same variant in multiple tumours available for analysis from the six patients in our group highlights the possibility of *IDH* somatic mosaicism; however, neither normal tissue nor blood were examined in the current report.

As is well-known, patients with Ollier disease and Maffucci syndrome have a marked risk of developing non-skeletal diverse malignancies, especially intracranial tumours of glial origin and ovarian sex cord-stromal tumours [2,24,25]. The genetic correlation among multiple tumours especially in extraosseous malignancies associated with the two disorders remains mysterious. We know that *IDH* mutations are well-detected in mostly enchondromas and spindle cell haemangiomas, and occasionally in other non-skeletal malignancies such as gliomas associated with Ollier disease, anaplastic astrocytoma and acute myeloid leukaemia in patients with Maffucci syndrome [26,27,28]. It seems that a mosaic pattern of *IDH* mutations could explain the pathogenesis of diverse tumours (multiple cartilaginous neoplasms, haemangiomas, gliomas or other uncommon extraosseous malignancies) in the same patient. However, juvenile granulosa cell tumours (JGCTs) are considered the most common reported extraskeletal tumours in patients with Ollier disease or Maffucci syndrome, which strongly suggests the possible connections between them [24,29]. However, there have been no previous reports on molecular features of JGCTs with Ollier disease or Maffucci syndrome. Other variants of ovarian sex cord-stromal tumour have rarely been reported. Only one case of ovarian cell-rich fibroma associated with Ollier disease has been reported to carry an *IDH1* mutation [30], but the detailed mutation position of it was not specified and the *IDH1* mutation status of the corresponding cartilage tumours is lacking.

In our group, the right ovarian tumour with Ollier disease in case one located ipsilaterally to the predominant skeletal dysplasia just as reported. Histologically, it was characterized by primitive undifferentiated ovate to elongated cells accompanied by stromal mucinous degeneration, and the mitotic figures were easy to find, but a pathological mitotic figure was absent. The tumour cells showed no immunoreaction with Desmin, MyoD1, Myogenin, SMA, S100, CD34 and BCOR, which were surrounded by reticular fibres. The only positive immunoreaction with α-inhibin suggested its ovarian sex cord-stroma origination, but this was insufficient for a conclusive diagnosis. Fortunately, an identical *IDH1 R132C* mutation was identified in both the ovarian tumour and the right femoral cartilage tumour. To the best of our knowledge, we reported, for the first time, that an identical *IDH1* mutation was represented in the ovarian tumour and corresponding cartilage tumour of Ollier disease. The demonstration of the *IDH1* mutation both in the ovarian tumour and cartilage tumour is of great significance. Theoretically, as the skeletal system and the gonads are both originated from the mesoderm, the involvement of the ovary is a manifestation of mesodermal dysplasia. Our result suggested that *IDH* mutation is pathognomonic for both the ovarian tumour and cartilage tumour in Ollier disease. 

In conclusion, *IDH* mutation may not be merely the initial cause of cartilage tumours; they are probably the intrinsic genetic link among multiple neoplastic lesions in the setting of somatic mosaicism. However, a heterozygotic IDH mutation alone is insufficient to induce gliomas or ovarian tumours in patients with Ollier disease and Maffucci syndrome. It may at least increase the tissue susceptibility of non-cartilaginous tumours’ formation. Future studies should demonstrate a causal effect and assess how these gene mutations lead to diverse neoplasms’ formation.

## Figures and Tables

**Table 1:** Clinicopathological data.

No	Sex	Onset Age	Age of Diagnosis	Locations	Treatment	Follow-Up (m)
1	F	2 yr	4 yr	Right upper femur, ilium, pubis, tibial metaphysis, right ovary right	Right upper femur biopsy and right ovarian mass resection	31
2	F	3 yr	18 yr	Right middle finger proximal segment, right third metacarpal bone, index finger proximal segment	Curettage of intraosseous lesions	40
3	M	7 yr	56 yr	Right little finger, ring finger, middle finger, right third, fourth, fifth metacarpal bone, right distal ulna	Resection of the fourth and fifth phalanges and half palms of the right hand	48
4	F	3 yr	20 yr	Left ulna, right index finger, little finger and ring finger; the right wrist and little finger subcutaneous soft tissue	Curettage of intraosseous lesions Resection of soft tissue mass	68
5	F	2 yr	36 yr	Bilateral scapula, sternum, left humerus, bilateral ilium, right acetabulum, left ischium, bilateral femur, right tibiofibula, right calcaneus; extensive subcutaneous nodules with calcification	Resection of right proximal tibia tumour Excision of subcutaneous mass in right leg	40
6	M	6 yr	33 yr	Multiple phalanges, metacarpals, ulna and radius in left hand, metatarsals, phalanges and distal tibia in left foot, and subcutaneous soft tissue nodules in the dorsum, sole and medial side of left foot	Curettage of short tubular lesions in left foot Resection of 2 subcutaneous nodules in the left foot	22

**Table 2:** Detection of *IDH1* mutation in 16 tumours.

No	Tumour Location	*IDH1* Mutation Results	*IDH2* Mutation Results
1	Right ovarian tumour	c.394C > T p.R132C	WT
	Endochondroma of right upper femur	c.394C > T p.R132C	WT
2	Endochondroma of proximal segment of right middle finger	c.394C > T p.R132C	WT
	Endochondroma of right third metacarpal bone	c.394C > T p.R132C	WT
	Endochondroma of proximal segment of index finger	WT	WT
3	Endochondroma of right little finger	c.395G > A p.R132H	WT
	Chondrosarcoma of right ring finger	c.395G > A p.R132H	WT
4	Subcutaneous haemangioma of right wrist	c.394C > T p.R132C	WT
	Subcutaneous haemangioma of right little finger	c.394C > T p.R132C	WT
	Endochondroma of right index finger	c.394C > T p.R132C	WT
	Endochondroma of right ring finger	WT	WT
5	Subcutaneous haemangioma of right leg	c.394C > T p.R132C	WT
	Chondrosarcoma of right proximal tibia	c.394C > T p.R132C	WT
6	Subcutaneous haemangioma of left medial malleolus	c.394C > T p.R132C	WT
	Endochondroma of left middle finger	c.394C > T p.R132C	WT
	Endochondroma of distal left tibia	WT	WT

## Data Availability

The data that support the findings of this study are available from the corresponding author, Zhiyan Liu, upon reasonable request.
